# Plexiform Fibromyxoma with MALAT1–GLI1 Fusion with Limited Myxoid Stroma, Aberrant KIT Expression, and Diffuse D2-40 Expression: A Case Report

**DOI:** 10.3390/diagnostics16060879

**Published:** 2026-03-16

**Authors:** Kotaro Watanabe, Kazuhito Tanaka, Kohei Ohkura, Kojiro Eto, Satoshi Ida, Kohei Yamashita, Yushi Kawakami, Keita Kai, Hidetaka Yamamoto, Yasuhito Tanaka, Masaaki Iwatsuki, Yoshiki Mikami

**Affiliations:** 1 Department of Diagnostic Pathology, Kumamoto University Hospital, Kumamoto 860-8556, Japan; 202m2110@st.kumamoto-u.ac.jp (K.W.); tanaka.kazuhito@kuh.kumamoto-u.ac.jp (K.T.); okura.kohei@kuh.kumamoto-u.ac.jp (K.O.); mika@kuhp.kyoto-u.ac.jp (Y.M.); 2 Department of Gastroenterological Surgery, Kumamoto University Hospital, Kumamoto 860-8556, Japan; etouetouetou@yahoo.co.jp (K.E.); s-ida@kumamoto-u.ac.jp (S.I.); k-yamashita@kumamoto-u.ac.jp (K.Y.); maiwa217@kumamoto-u.ac.jp (M.I.); 3 Department of Gastroenterology and Hepatology, Kumamoto University Hospital, Kumamoto 860-8556, Japan; yuushi0202@yahoo.co.jp (Y.K.); ytanaka@kumamoto-u.ac.jp (Y.T.); 4 Department of Pathology, Graduate School of Medicine, Dentistry & Pharmaceutical Science, Okayama University, Okayama 700-8558, Japan; hideyamamoto@okayama-u.ac.jp

**Keywords:** plexiform fibromyxoma, stomach, D2-40, MALAT1-GLI1 fusion, variant morphology

## Abstract

**Background and Clinical Significance**: Plexiform fibromyxoma (PFM) is a rare benign gastric mesenchymal neoplasm characterized by multinodular plexiform growth of bland spindle cells in a myxoid or fibromyxoid stroma. We report a case of the cellular form of PFM with limited myxoid stroma and aberrant KIT expression, resulting in diagnostic difficulty by biopsy. **Case Presentation**: A 59-year-old woman presented with a slowly enlarging 15 mm gastric antral submucosal tumor. A resected specimen by laparoscopic and endoscopic cooperative surgery revealed spindle cell proliferation forming plexiform nodules with a myxoid background in limited areas. Positive immunoreactivity of a subset of spindle cells for KIT suggested a diagnosis of gastrointestinal stromal tumor (GIST), although DOG1 was negative. In addition, diffuse staining for CD10, smooth muscle actin, and D2-40 was confusing. MALAT1::GLI1 fusion was detected by next-generation sequencing analysis. Consequently, a diagnosis of PFM was established. **Conclusions**: This case expands the morphologic and immunophenotypic spectrum of PFM and indicates the possible diagnostic utility and biological significance of D2-40 expression. Although molecular confirmation of MALAT1::GLI1 fusion is definitive for the diagnosis of PFM, the findings of the present case may aid diagnosis in challenging cases that mimic GIST.

## 1. Introduction

Plexiform fibromyxoma (PFM) is a rare benign gastric mesenchymal neoplasm first described by Takahashi et al. in 2007 as “plexiform angiomyxoid myofibroblastic tumor” [1]. The term “plexiform fibromyxoma” was proposed by Miettinen et al. in 2009 [2] and then PFM was officially listed as a distinct type of gastric mesenchymal neoplasm by the World Health Organization (WHO) in 2010 [3]. In the English literature, more than 130 cases have been reported [4]. PFM typically presents a multinodular plexiform growth of bland spindle cells embedded in a myxoid or fibromyxoid stroma with increased vascularity [3]. Molecular studies have identified fusions of recurrent metastasis-associated lung adenocarcinoma transcript 1 (MALAT1)::glioma-associated oncogene homolog 1 (GLI1) that drive GLI1 overexpression in a subset of PFM [5,6]. Here, we present a case of a cellular form of PFM with limited myxoid matrix, showing diffuse D2-40 expression, resulting in a diagnostic challenge.

## 2. Case Presentation

A 59-year-old woman was referred to our hospital for evaluation of a gastric submucosal tumor at the antrum that was detected at a medical checkup, and which had been under surveillance for several years. She has no remarkable past medical history or family history. Serial endoscopy revealed an enlargement of the submucosal tumor from 10 mm to 15 mm over the preceding 12 months. The laboratory data were unremarkable. Contrast-enhanced computed tomography demonstrated a well-circumscribed exophytic mass along the greater curvature and no evidence of a metastatic lesion was noted. Microscopic examination of an endoscopic biopsy specimen suggested a benign or low-grade spindle cell neoplasm, i.e., gastrointestinal stromal tumor (GIST) or a myopericytomatous tumor, but failed to establish a definitive diagnosis. Local resection of the stomach was performed using laparoscopic and endoscopic cooperative surgery. The postoperative course was uneventful and the patient was discharged on postoperative day 7. No evidence of a recurrent tumor was identified at the time of writing (8 months after surgery).

### Pathological Findings

**Figure 1:**
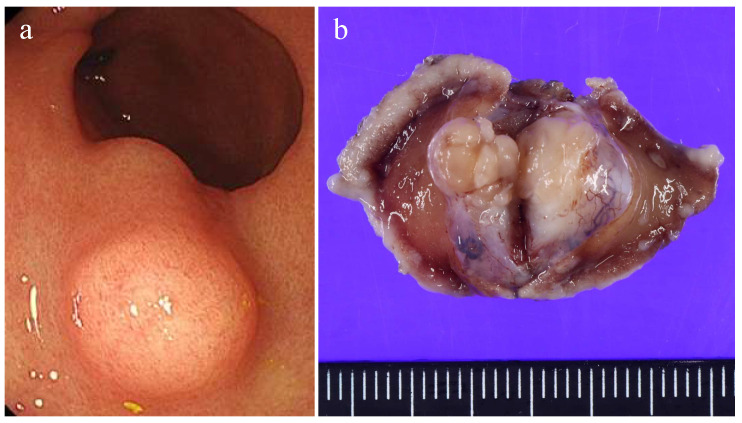
(**a**) Endoscopic appearance. The submucosal tumor covered by non-neoplastic mucosa is shown. (**b**) Gross appearance of a formalin-fixed specimen.

Gross examination revealed a lobulated, solid submucosal tumor, measuring 15 × 11 × 10 mm, presenting as a protruding lesion (Figure 1). The cut surface was homogeneous without hemorrhage or necrosis.

**Figure 2:**
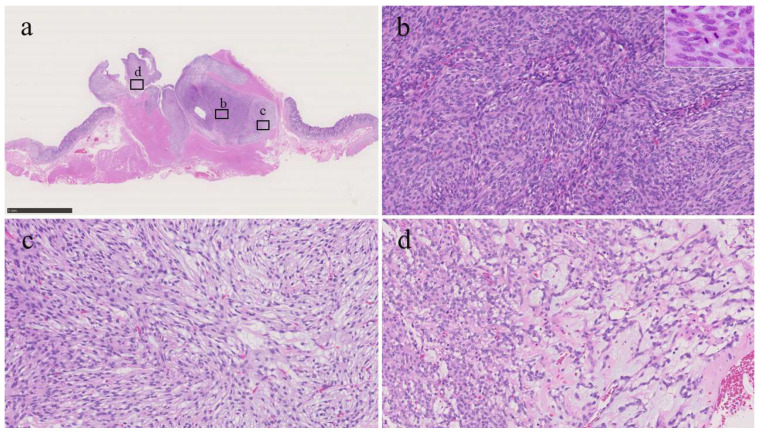
Histological findings of the tumor. (**a**) Loupe image. The tumor proliferated with a plexiform appearance and tongue-like extension into the muscularis propria (bar = 5 mm). (**b**) The area of high-cellularity without mucinous stroma (original magnification: ×200). Inset shows a mitotic area (×400). (**c**) An area of intermediate-cellularity with a small amount of fibromyxoid stoma (×200). (**d**) Area showing focal myxoid stroma (×200).

Histologically, the tumor had a plexiform appearance and a tongue-like extension into the muscularis propria, forming a lobulated mass (Figure 2a), which was composed of bland-looking spindle cells without significant nuclear atypia, arranged in fascicular or streaming patterns with only limited fibromyxoid or myxoid stroma (Figure 2b–d). The mitotic count was 11 per 50 high-power fields (HPFs). Mitoses were predominantly observed in the hypercellular areas (Figure 2b inset); no atypical mitotic figures were identified. No necrosis or lymphovascular invasion was identified, and all surgical margins were negative.

**Figure 3:**
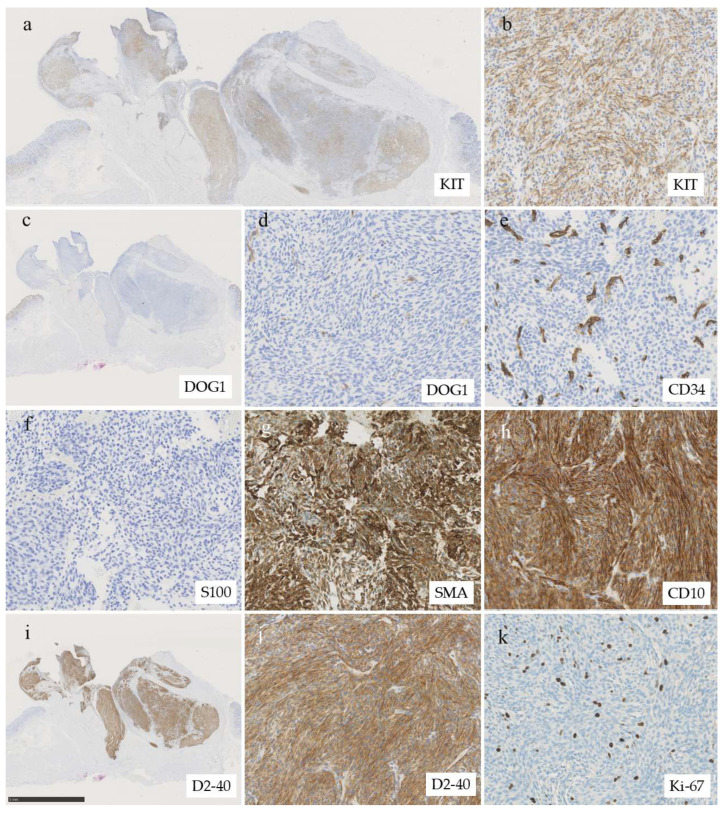
Immunohistochemical analysis. (**a**) Loupe image of immunohistochemistry of KIT (CD117). Tumor cells show patchy (multifocal) expression of KIT (CD117). (**b**) High-power view of positive area of KIT (original magnification: ×200). (**c**) Loupe image of immunohistochemistry of DOG1. Tumor cells are negative for DOG1. (**d**) High power view of DOG1 immunohistochemistry (×200), (**e**) Immunohistochemistry of CD34 (×200). The tumor cells are negative for CD34 while stromal vessels positive for CD34. Tumor cells are negative for (**f**) S100 protein (×200) and are diffusely positive for (**g**) SMA (×200) and (**h**) CD10 (×200). (**i**) Loupe image of D2-40 immunohistochemistry (bar = 5 mm). The entire tumor expressed D2-40. (**j**) High power view of D2-40 immunohistochemistry (×200). (**k**) The Ki-67 labeling index was approximately 5% (×200).

Immunohistochemical analysis indicated the tumor cells were focally positive for KIT (CD117) (Figure 3a,b), but negative for DOG1 (Figure 3c,d), making a diagnosis of GIST unlikely. DOG1 is considered a sensitive and specific marker for GIST, with both sensitivity and specificity reported to exceed 95%, regardless of CD117 expression and independent of KIT or platelet-derived growth factor receptor α (PDGFRA) mutation status [7,8]. Succinate dehydrogenase (SDH)-deficient GIST was also excluded because SDHB expression was retained. Other differential diagnoses, including schwannoma, leiomyoma, glomus tumor, gastroblastoma, inflammatory myofibroblastic tumor (IMT), inflammatory fibroid polyp, and perivascular epithelioid cell tumor (PEComa), were also excluded based on a combination of morphology and results of extensive immunohistochemical workup (Table 1). The tumor cells stained negative for cytokeratin (AE1/AE3), CD34 (Figure 3e), desmin, h-caldesmon, synaptophysin, S100 protein (Figure 3f), HMB45, Melan A, and anaplastic lymphoma kinase (ALK). Of note, the tumor cells stained positive for smooth muscle actin (SMA), CD10, and D2-40 (Figure 3g–j). Diffuse D2-40 expression made mesothelioma and follicular dendritic cell sarcoma diagnostic concerns, but mesothelial markers (calretinin and WT-1) and follicular dendritic cell markers (CD21, CD23, and ICOS) were negative. The Ki-67 labeling index was 5.2% (91/1736) at the hot spot (Figure 3k). Because the case remained diagnostically challenging, on this case we consulted with one of the co-authors who is a recognized pathologist in Japan (H.Y.). Based on the low-power finding of plexiform architecture (Figure 2a) and the presence of focal myxoid stroma, PFM emerged as a diagnostic consideration.

Finally, because the diagnosis of PFM remained a serious diagnostic consideration, analysis using a next-generation sequencing panel (Archer FusionPlex Sarcoma Panel) supported by the consultation system of the National Cancer Center (Tokyo, Japan) was requested. RNA fusion analysis identified a MALAT1::GLI1 fusion, involving MALAT1 exon 1 (NR_002819.4) and GLI1 exon 5 (NM_005269.3), which passed strong-evidence filters and was not flagged as an artifact. The fusion was supported by 4555 reads/84 unique start sites (MALAT1→GLI1) and 1408 reads/145 unique start sites (GLI1→MALAT1); a minor additional isoform (GLI1 intron 5 to MALAT1 exon 1) was also detected (14 reads/10 start sites). This revealed a MALAT1::GLI1 fusion, indicating a diagnosis of PFM. Gastroblastoma was also considered because it can share the MALAT1::GLI1 fusion; however, our tumor lacked a biphasic epithelial component and was negative for cytokeratin (AE1/AE3), arguing strongly against gastroblastoma. Although the mitotic activity appeared higher compared with that in a previous series of PFM cases, our case was considered not to fit the morphological characteristics of a malignant epithelioid tumor with GLI1 rearrangement [4,9,10].

## 3. Discussion

PFM is characterized by a distinctive plexiform growth pattern composed of bland spindle to stellate cells embedded within a myxoid or fibromyxoid stroma, often accompanied by increased vascularity [3]. This morphological appearance frequently overlaps with other gastrointestinal stromal tumors, such as myxoid GIST, nerve sheath tumor, smooth muscle tumor, glomus tumor, gastroblastoma, IMT, and inflammatory fibroid polyp [2,4,5]. PFM presents a significant diagnostic challenge because of its rarity and the variety of mimics, particularly when using small biopsy samples. In the current case, cellular morphology with only limited myxoid stroma and focal KIT expression was also a source of diagnostic confusion.

The molecular features of PFM have been investigated [4,5]. PFM lacks KIT, platelet-derived growth factor-A, and SDH gene mutations that are definitional alterations of GISTs. In 2016, Spans et al. [6] reported that a minor subset of PFM (3/16 cases, 18%) harbored MALAT1::GLI1 gene fusions. Banerjee et al. [11] reported PTCH1 inactivation via gene or chromosomal deletion in 2 of 8 cases of PFM (5 cases used for NGS analysis).

It should be noted that the MALAT1::GLI1 fusion has been demonstrated in other tumors, including gastroblastoma and malignant epithelioid tumor with GLI1 rearrangement, which is a recently described epithelioid neoplasm of soft tissue, typically with S100 protein expression and variable expression of cytokeratin [9]. In the gastrointestinal tract, only one case involving the jejunum has been reported [10]. Although the mitotic activity and Ki-67 labeling index in our case were relatively higher (11/50 HPF and 5.2%, respectively) compared with those in a previous series of PFM cases (median: 1/50 HPF and less than 2%, respectively) [2,11], the immunophenotype, i.e., negative staining for S100 and cytokeratin, and the absence of epithelioid morphology, distinct nuclear atypia, necrosis, and lymphovascular space invasion justified the diagnosis of PFM. Although the follow-up period was limited, there was no evidence of recurrent disease 8 months after resection.

A diagnosis of PFM depends on morphology and immunophenotyping to exclude other mesenchymal tumors, especially GIST. To date, no specific IHC marker for the diagnosis of PFM has been established. The utility of GLI1 IHC has been questioned [5], because MALAT1::GLI1 fusion has been identified in a minor subset of PFM. Hu et al. [12] investigated the immunohistochemical profile of 10 cases of PFM and demonstrated that all cases were positive for vimentin and SMA, and some cases were positive for CD10 (5/10), desmin (5/10), h-caldesmon (6/10), and progesterone receptor (6/10), whereas CD34, S100 protein, estrogen receptor, anaplastic lymphoma kinase (ALK), KIT, and DOG1 were all negative.

The current case highlights diffuse D2-40 expression in PFM. D2-40 is a monoclonal antibody directed against podoplanin, a transmembrane glycoprotein involved in lymphangiogenesis, cell migration, and epithelial–mesenchymal transition [13]. Our literature search revealed only three single case reports of PFM referring to D2-40 expression. Of these three cases, one showed diffuse expression [14], whereas the remaining two cases were negative [15,16], although, unfortunately, these three case reports did not investigate MALAT1::GLI1 fusion. D2-40 IHC is a commonly used diagnostic tool among pathologists to identify lymphatic vessels, and tumors that could be the differential diagnosis of PFM were usually negative for this marker (Table 1). Future studies should examine the utility of D2-40 involving a series of PFM cases with confirmed MALAT1::GLI1 fusion.

## 4. Conclusions

PFM can be a diagnostic challenge because of the absence of or a limited myxoid background and patchy non-specific KIT expression. Diffuse D2-40 expression in the current case contributes to diagnostic confusion and potential pitfalls because D2-40 is not considered as an established surrogate marker for MALAT1::GLI1 fusion at present. In this regard, the spectrum of morphology, as represented by the current case, is an essential component for avoiding diagnostic misinterpretations. Further investigations are expected to clarify the frequency and significance of D2-40 expression in PFM.

## Figures and Tables

**Table 1:** Possible differential diagnoses for plexiform fibromyxoma.

Tumors	Typical Morphology	Useful Immunohistochemistry	Molecular Alteration
Plexiform fibromyxoma	Multinodular and plexiform low-power architecture; uniform spindle cells in a myxoid, fibromyxoid, or collagenous stroma	None (usually positive for SMA, occasionally positive for CD10, desmin, caldesmon, D2-40)	MALAT1::GLI1, PTCH1 inactivation
GIST	Intramural, submucosal, or subserosal mass; spindle cell, epithelial, or mixed morphology	Positive for KIT, DOG-1	KIT, PDGFRA
SDH-deficient GIST	Characteristic epithelioid morphology and typically multinodular with plexiform mural involvement	Positive for KIT, DOG-1, loss of SDHB	SDHB, No KIT or PDGFRA mutation
Leiomyoma	Fascicles of spindle cells with eosinophilic cytoplasm	Positive for desmin, SMA	
Schwannoma	Well-circumscribed mass; areas of cellular and hypocellular spindle cells	Positive for S100	NF1, NF2
Perineurioma	Uniform spindle cells, most are small colonic polyps	Positive for EMA, Negative for S100	NF2, BRAF (associated serrated polyp)
Glomus tumor	Round glomoid cells; sometimes plexiform growth	Positive for SMA, collagen IV	NOTCH, BRAF
PEComa	Epithelioid and/or spindle cells with granular eosinophilic-to-clear cytoplasm	Positive for smooth muscle (desmin, SMA) and melanocytic (HMB45, melan-A, PNL2, MITF, tyrosinase) markers	TSC1/2, TFE3 (minority)
Inflammatory fibroid polyp	Hypocellular, with short spindled-to-stellate cells, infiltration of eosinophils and lymphocytes.	Positive for CD34, PDGFRA	PDGFRA
Inflammatory myofibroblastic tumor	Loose fascicles of spindle cells without pleomorphism, infiltration of lymphocytes and plasma cells	Positive for SMA, ALK	ALK, ROS1
Solitary fibrous tumor	Fascicles of uniform ovoid-to-spindle cells, staghorn vessels	Positive for CD34, STAT6	NAB2::STAT6
Synovial sarcoma	Spindle cell (monophasic) or spindle cell with epithelioid-to-glandular (biphasic)	None (focal positivity for keratin and EMA)	SS18::SSX1/2/4
Gastroblastoma	Biphasic with spindle cells and epithelial cells	Epithelial cells are positive for CK, and spindle and epithelial cells are focally positive for CD10 and CD56	MALAT1::GLI1
Malignant epithelioid tumor with GLI1 rearrangement	Epithelioid, ovoid, round to spindle	Occasionally positive for S100, a subset case focally positive for CK	MALAT1::GLI1

SMA, smooth muscle actin; CD, cluster of differentiation; MALAT1, metastasis-associated lung adenocarcinoma transcript 1; GLI1, glioma-associated oncogene homolog 1; PTCH1, patched 1; GIST, gastrointestinal stromal tumor; DOG-1, discovered on GIST-1; PDGFRA, platelet-derived growth factor receptor alpha; SDH, succinate dehydrogenase; SDHB, succinate dehydrogenase complex iron sulfur subunit B; NF, neurofibromin; EMA, epithelial membrane antigen; PEComa, perivascular epithelioid cell tumor; HMB45, human melanoma black 45; melan-A, melanoma antigen recognized by T cells 1; MITF, microphthalmia-associated transcription factor; TFE3, transcription factor binding to IGHM enhancer 3; TSC, tuberous sclerosis complex; ALK, anaplastic lymphoma kinase; STAT6, signal transducer and activator of transcription 6; NAB2, NGFI-A binding protein 2; CK, cytokeratin.

## Data Availability

The original contributions presented in this study are included in the article. Further inquiries can be directed to the corresponding author.

## Abbreviations

The following abbreviations are used in this manuscript:

PFM	Plexiform fibromyxoma
IHC	Immunohistochemistry
DOG1	Discovered on gastrointestinal stromal tumor 1
SMA	Smooth muscle actin
SDHB	Succinate dehydrogenase subunit B
MALAT1	Metastasis-associated lung adenocarcinoma transcript 1
GLI1	Glioma-associated oncogene homolog 1
GIST	Gastrointestinal stromal tumor
HE	Hematoxylin-eosin
SDH	Succinate dehydrogenase
HPF	High-power fields
IMT	Inflammatory myofibroblastic tumor
PEComa	Perivascular epithelioid cell tumor
CD	Cluster of differentiation
ICOS	Inducible T-cell co-stimulator
NGS	Next-generation sequencing
WT-1	Wilms tumor 1

## References

[1] Takahashi Y., Shimizu S., Ishida T., Aita K., Toida S., Fukusato T., Mori S. (2007). Plexiform angiomyxoid myofibroblastic tumor of the stomach. Am. J. Surg. Pathol..

[2] Miettinen M., Makhlouf H.R., Sobin L.H., Lasota J. (2009). Plexiform fibromyxoma: A distinctive benign gastric antral neoplasm not to be confused with a myxoid GIST. Am. J. Surg. Pathol..

[3] Miettinen M., Fletcher C.D., Kindblom L.G., Tsui W.M., Bosman F.T., Carneiro F., Hruban R., Teise N.D. (2010). Mesenchymal tumors of the stomach. WHO Classification of Tumours of the Digestive System.

[4] Arslan M.E., Li H., Fu Z., Jennings T.A., Lee H. (2021). Plexiform fibromyxoma: Review of rare mesenchymal gastric neoplasm and its differential diagnosis. World J. Gastrointest. Oncol..

[5] Szczepanski J., Westerhoff M., Schechter S. (2025). Plexiform Fibromyxoma: A Review and Discussion of the Differential Diagnosis of Gastrointestinal Mesenchymal Tumors. Arch. Pathol. Lab. Med..

[6] Spans L., Fletcher C.D., Antonescu C.R., Rouquette A., Coindre J.M., Sciot R., Debiec-Rychter M. (2016). Recurrent *MALAT1-GLI1* oncogenic fusion and *GLI1* up-regulation define a subset of plexiform fibromyxoma. J. Pathol..

[7] Miettinen M., Wang Z.F., Lasota J. (2009). DOG1 antibody in the differential diagnosis of gastrointestinal stromal tumors: A study of 1840 cases. Am. J. Surg. Pathol..

[8] Wu C.-E., Tzen C.-Y., Wang S.-Y., Yeh C.-N. (2019). Clinical Diagnosis of Gastrointestinal Stromal Tumor (GIST): From the Molecular Genetic Point of View. Cancers.

[9] Antonescu C.R., Agaram N.P., Sung Y.S., Zhang L., Swanson D., Dickson B.C. (2018). A distinct malignant epithelioid neoplasm with GLI1 gene rearrangements, frequent S100 protein expression, and metastatic potential: Expanding the spectrum of pathologic entities with ACTB/MALAT1/PTCH1-GLI1 fusions. Am. J. Surg. Pathol..

[10] Prall O.W.J., McEvoy C.R.E., Byrne D.J., Iravani A., Browning J., Choong D.Y., Yellapu B., O’hAire S., Smith K., Luen S.J. (2020). A malignant neoplasm from the jejunum with a MALAT1-GLI1 fusion and 26-year survival history. Int. J. Surg. Pathol..

[11] Banerjee S., Corless C.L., Miettinen M.M., Noh S., Ustoy R., Davis J.L., Tang C.M., Yebra M., Burgoyne A.M., Sicklick J.K. (2019). Loss of the PTCH1 tumor suppressor defines a new subset of plexiform fibromyxoma. J. Transl. Med..

[12] Hu G., Chen H., Liu Q., Wei J., Feng Y., Fu W., Zhang M., Wu H., Gu B., Ren J. (2017). Plexiform fibromyxoma of the stomach: A clinicopathological study of 10 cases. Int. J. Clin. Exp. Pathol..

[13] Ordóñez N.G. (2006). Podoplanin: A novel diagnostic immunohistochemical marker. Adv. Anat. Pathol..

[14] Ebi M., Nagao K., Sugiyama T., Yamamoto K., Saito T., Kurahashi S., Yamaguchi Y., Adachi K., Tamura Y., Izawa S. (2022). Gastric plexiform fibromyxoma resected using non-exposed endoscopic wall-inversion surgery: A case report. Case Rep. Gastroenterol..

[15] Kane J.R., Lewis N., Lin R., Villa C., Larson A., Wayne J.D., Yeldandi A.V., Laskin W.B. (2016). Plexiform fibromyxoma with cotyledon-like serosal growth: A case report of a rare gastric tumor and review of the literature. Oncol. Lett..

[16] Tang J., Liu F. (2020). Plexiform fibromyxoma: A rare mesenchymal tumor found in the esophagus. Am. J. Gastroenterol..

